# Peripheral and posterior pole retinal changes in highly myopic Chinese children and adolescents: Retinal changes in Chinese children and adolescents

**DOI:** 10.1186/s12886-024-03328-6

**Published:** 2024-02-13

**Authors:** Wenli Zhang, Fan Yang, Shirong Chen, Tingkun Shi

**Affiliations:** Joint Shantou International Eye Center, Shantou University & the Chinese University of Hong Kong, North Dongxia Road, 515041 Shantou, Guangdong China

**Keywords:** Chinese adolescents, High myopia, Prevalence, Peripheral and posterior retinal changes

## Abstract

**Purpose:**

This study was conducted to evaluate the prevalence and related factors of peripheral and posterior pole retinal changes in highly myopic Chinese children and adolescents.

**Methods:**

A hospital-based cross-sectional study was designed. A total of 120 subjects with high myopia were recruited and underwent cycloplegic refraction, dilated fundus examination, and optical coherence tomography. A statistical analysis was performed to evaluate the factors associated with peripheral and posterior pole retinal changes.

**Results:**

The mean spherical equivalent refraction of the subjects was − 8.74 ± 2.86 D, and the mean age was 11.45 ± 3.02 years. Snowflake retinal degeneration (27.5%), white without pressure (27.5%), snail-track degeneration (15%), and lattice degeneration (15%) were the most common peripheral retinal changes, while tessellated fundus (84.17%), optic nerve crescents (78.3%), and posterior staphyloma (11.7%) were the most common posterior changes. Subjects with peripheral changes were significantly older, with thinner choroids (OR = 1.194, 95% CI: 1.045–1.363, *p* = 0.009; OR = 0.993, 95% CI: 0.987–0.999, *p* = 0.022, respectively). Optic nerve crescents, tessellated fundus, and posterior scleral staphyloma were all associated with thin choroids (OR = 0.990, 95% CI: 0.983–0.997, *p* = 0.008; OR = 0.983, 95% CI: 0.974–0.991, *p* < 0.001; OR = 0.974, 95% CI: 0.960–0.987, *p* < 0.001, respectively).

**Conclusion:**

A substantial proportion of the subjects had peripheral and posterior retinal changes. An increased risk of retinal changes was associated with high degrees of myopia, long axial lengths, thin choroids, and older ages among 7–16-year-old individuals.

## Introduction

High myopia is not only an optical problem but also comes with a high risk of ocular complications, including peripheral retinal changes (e.g., retinal breaks or holes and lattice degeneration) and posterior retinal changes (e.g., posterior staphyloma, optic nerve crescents, lacquer cracks, and Fuchs spot) [1, 2]. These are critical risk factors for developing rhegmatogenous retinal detachment (RRD) and myopic maculopathy [3–5]. Therefore, recognizing the early signs of retinal changes and related risk factors in childhood is critical for the early prediction, diagnosis, and subsequent treatment of severe complications.

Several studies have demonstrated that an increased prevalence of peripheral retinal changes is associated with high myopia and increased axial length in adults [2, 6–8]. However, the risk factors for retinal degeneration in children and adolescents have not yet been well characterized. Compared to adults, children are less likely to report symptoms associated with RRD and often have severe proliferative vitreoretinopathy. Furthermore, vitreoretinal surgery in children is complex and has a low success rate [9]. Considering this, the early detection of risk factors and prompt interventions have become crucial for the effective treatment of RRD.

We performed this study to determine the prevalence of peripheral and posterior pole retinal changes and to explore the related risk factors among children and adolescents. We hope that our results provide helpful information to the public on this ocular issue.

## Subjects and methods

A hospital-based cross-sectional study design was adopted. Highly myopic children and adolescents were consecutively recruited from the myopia prevention and control clinic at the Joint Shantou International Eye Center (JSIEC) between January 2020 and December 2021. The subjects who met the following criteria were included in the study: ethnic Chinese, 7–16 years of age, spherical equivalent refractive error less than − 5 D for children aged 7–12 years and less than − 6 D for those aged ≥ 13 years [10]. Subjects with a history of retinopathy of prematurity, Stickler syndrome, ocular trauma, uveitis, orthokeratology treatment, or eyes with media opacities causing unclear fundus examinations were excluded. If a subject had high bilateral myopia, only the right eye was considered. The ethics committee at JSIEC approved the study, and the tenets of the Declaration of Helsinki were followed. Informed consent was obtained from the subjects or their guardians.

Each subject underwent a comprehensive ocular examination, which included measurements of best-corrected visual acuity (BCVA) using the Snellen chart, intraocular pressure (IOP) using a noncontact tonometer (CT-800, Topcon, Japan), cycloplegic autorefraction using KR-800 (Topcon, Japan), and axial length using an IOL Master (700, ZEISS, Germany), as well as a slit-lamp biomicroscope examination. Three drops of 0.5% tropicamide phenylephrine were instilled in both eyes 5 min apart, followed by cycloplegic retinoscopy and subjective refraction 30 min later to define the refractive status of each participant. Ultrawide-field fundus photography (using Daytona P200T, Optomap, Germany) was routinely performed. Standard dilated fundus examinations were conducted by a retinal specialist (TS) with an indirect ophthalmoscope and a Volk lens biomicroscope. If retinal changes were identified in the binocular indirect ophthalmoscope but not in the ultrawide field fundus photograph, supplementary photographs of the retinal changes were taken with a digital camera. All fundus examination findings, including peripheral and posterior pole retinal changes, were recorded using a predesigned form. Swept-source optical coherence tomography (SS-OCT; using DRI OCT-1 Atlantis, Topcon, Japan) was performed according to the 12 mm line scan protocol with an average of 128 consecutive, overlapping single B-scan OCT images, as we’ve previously reported [11]. We used OCT scans to evaluate the choroidal thickness and retinal thickness of the posterior pole and to identify slight posterior staphyloma.

**Fig. 1:**
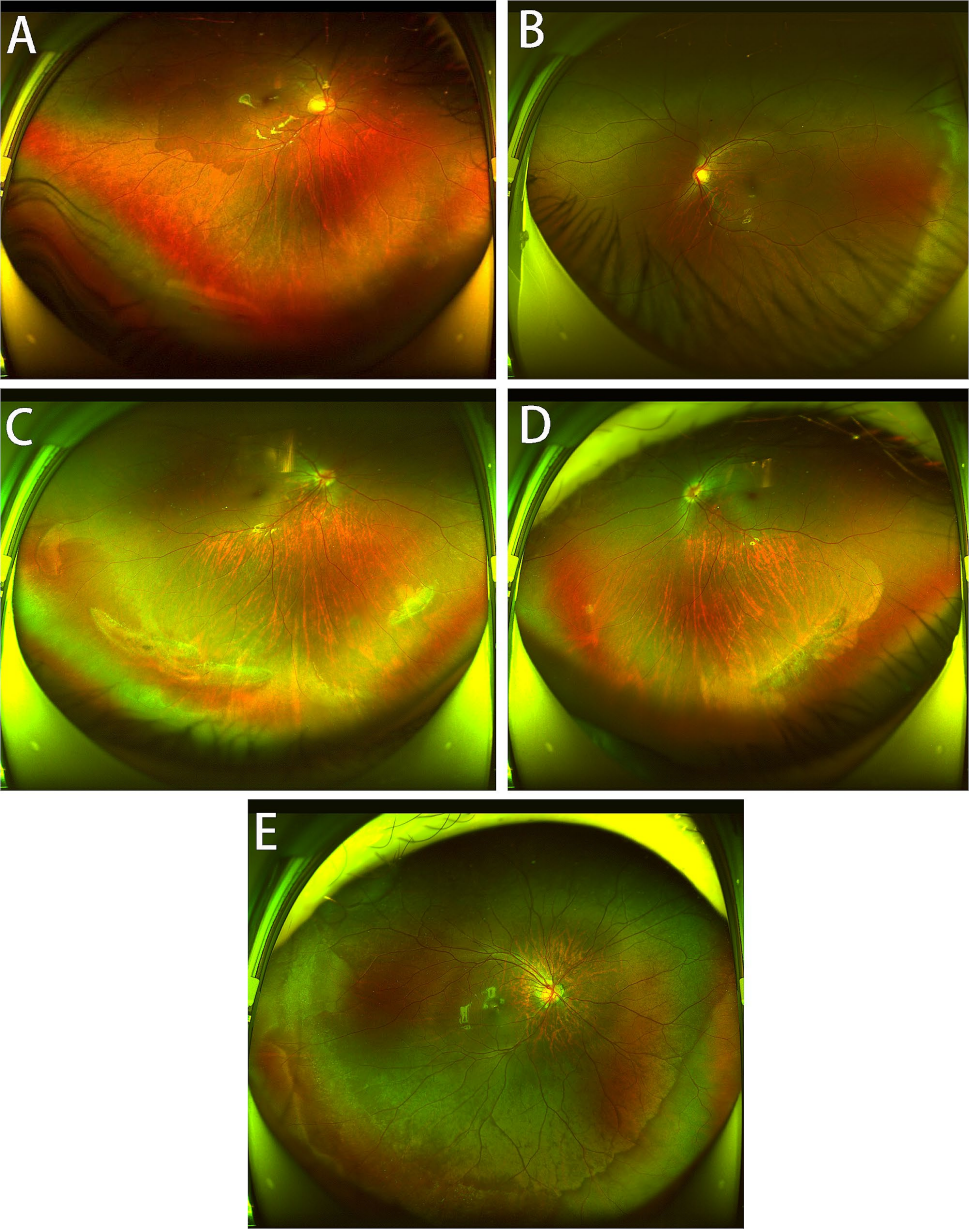
Wild-field fundus photograph showing a tessellate fundus with different types of peripheral retinal changes; **(A)** retinal hole (inferior peripheral), **(B)** white without pressure, **(C)** snail-track degeneration, **(D)** lattice degeneration, **(E)** snowflake degeneration

Peripheral and posterior pole retinal changes were defined as follows: lattice degeneration involved spindle-shaped areas of retinal thinning; white without pressure denoted retinal areas with a translucent white-gray appearance; snail-track degeneration was characterized by sharply demarcated bands of tightly packed “snowflakes” that gave the peripheral retina a white frost-like appearance (Fig. 1); optic nerve crescents appeared as whitish areas adjacent to the optic nerve head, where the scleral could be viewed directly due to a loss of deep retinal layers; posterior staphyloma referred to an outpouching of a circumscribed region of the posterior fundus; and tessellated fundus were well-defined choroidal vessels that could be observed clearly around the fovea and the arcade vessels.

The statistical analysis was conducted using SPSS v19.0 (SPSS, Chicago, IL, USA). Continuous variables were recorded as mean ± standard deviation (SD) values and ranges. The BCVA was transformed to the logarithm of the minimum angle of resolution (Log MAR) for further analysis. Pearson’s correlation was used to evaluate the relationship between axial length and spherical equivalent refractive error. Independent t-tests and analyses of variance were used to compare normally continuous data. Binary logistic regression analyses were performed to investigate the factors associated with retinal changes, using the retinal changes as dependent variables and various baseline characteristics as covariates and adjusting for age and gender, where appropriate. The level of *p* < 0.05 was assumed to have statistical significance.

## Results

### Subjects and demographics

A total of 120 subjects were recruited, including 63 (52.5%) girls and 57 (47.5) boys (Table 1). Among them, 80 (66.7%) subjects had high bilateral myopia, while 40 (33.3%) had unilateral high myopia. By default, the right eyes of subjects with high bilateral myopia were included for further analysis. For unilateral cases, only highly myopic eyes were included. The baseline demographic and ocular biometry parameters are given in Table 1. The mean age of the subjects was 11.45 ± 3.02 years. The mean spherical equivalent refractive error was − 8.74 ± 2.86 D. The mean axial length of the eyes was 26.68 ± 1.40 mm. A statistically significant correlation was found between spherical equivalent refractive error and axial length (Person’s correlation, *r* = -0.638, *p* < 0.01).

**Table 1:** Clinical characteristics of the study subjects

Number of eyes	120	
Gender (Female: Male)	63/57	
Age (Year)	11.45 ± 3.02	
BMI, Body mass index	17.66 ± 3.11	
Spherical equivalent(D)	-8.74 ± 2.86	
BCVA (LogMAR)	0.20 ± 0.31	
IOP (mmHg)	17.42 ± 2.72	
Family history	24	
Axial length (mm)	26.68 ± 1.40	
C/D ratios	0.31 ± 0.08	
Central foveal thickness (µm)	212.73 ± 20.83	
Choroidal thickness (µm)	191.08 ± 64.91	

### Frequencies of retinal changes

The frequencies of the retinal changes found are shown in Table 2. More than half of the eyes had tessellated fundus (84.17%) and optic nerve crescents (78.33%), 27% of the eyes were white without pressure and exhibited frost degeneration, and 15% showed lattice degeneration and snail-track degeneration. The following retinal changes were also found: posterior staphyloma in 14 eyes (11.67%), retinal holes in two eyes (1.67%), retinal thinning in one eye (0.83%), and peripheral pigmentary degeneration in one eye (0.83%).

**Table 2:** Number of eyes with retinal changes

Type of peripheral retinal lesion	Number of eyes	Percentage (%)
Lattice degeneration	18	15.00%
White without pressure	33	27.50%
Snail-track degeneration	18	15.00%
Frost degeneration	33	27.50%
Retinal hole	2	1.67%
Microcystoid degeneration	0	0.00%
Retinal thinning	1	0.83%
Peripheral pigmentary degeneration	1	0.83%
Any peripheral retinal changes	65	54.17%
Optic nerve crescent	94	78.33%
Posterior staphyloma	14	11.67%
Tessellated fundus	101	84.17%
Any retinal changes	107	89.17%

### Presence of posterior Pole retinal changes

Of the 120 eyes (11.66%), 14 had posterior staphyloma, 94 (78.33%) had optic nerve crescents, and 101 (84.17%) had tessellated fundus. No chorioretinal atrophy, Fuchs spots, or lacquer crack lesions were found. The mean values of the parameters in eyes with and without posterior retinal changes are summarized in Table 3. Subjects with optic nerve crescents had higher spherical equivalent errors and thinner choroids than subjects without optic nerve crescents. Further, the independent t-tests showed that eyes with posterior staphyloma had a significantly higher mean spherical equivalent refractive error, longer axial lengths, and thinner choroids. Similar results were found in the tessellated fundus groups. A binary logistic regression analysis revealed that, after controlling for axial length, choroidal thickness was significantly associated with all three of these posterior pole retinal changes (Table 4).

**Table 3:** Mean values of parameters in eyes with and without posterior retinal changes

	Optic nerve crescent			Posterior scleral staphyloma			Tessellate fundus		
Parameters	with n = 94	without n = 26	*P value*	with n = 14	without n = 26	*P value*	with n = 101	without n = 19	*P value*
Gender (Female: Male)	52/42	11/15	0.24	7/7	56/50	0.84	54/47	9/10	0.95
Age (Year)	11.44 ± 3.09	11.50 ± 2.77	0.92	10.64 ± 3.17	11.56 ± 2.99	0.29	11.45 ± 3.13	11.47 ± 2.41	0.97
BMI, Body mass index	17.66 ± 3.17	17.68 ± 2.95	0.97	16.57 ± 2.65	17.81 ± 3.15	0.16	17.33 ± 2.79	19.42 ± 4.06	0.007*
Spherical equivalent(D)	-9.00 ± 3.08	-7.81 ± 1.55	< 0.01	-11.96 ± 3.43	-8.32 ± 2.50	< 0.01*	-9.05 ± 2.97	-7.11 ± 1.24	0.006*
BCVA (LogMAR)	0.21 ± 0.32	0.16 ± 0.27	0.48	0.18 ± 0.29	0.17 ± 0.26	0.06	0.23 ± 0.33	0.07 ± 0.09	0.04*
IOP (mmHg)	17.36 ± 2.71	17.62 ± 2.80	0.68	16.59 ± 2.65	17.50 ± 2.71	0.36	17.34 ± 2.68	17.84 ± 2.96	0.46
Family history	20	4	0.51	4	20	0.48	19	5	0.34
Axial length (mm)	26.75 ± 1.45	26.40 ± 1.16	0.26	28.11 ± 2.06	26.49 ± 1.17	0.01*	26.79 ± 1.43	26.07 ± 1.03	0.04*
C/D ratios	0.31 ± 0.07	0.32 ± 0.11	0.6	0.27 ± 0.08	0.32 ± 0.08	0.06	0.31 ± 0.08	0.32 ± 0.08	0.74
ACD	3.80 ± 0.25	3.69 ± 0.25	0.06	3.69 ± 0.17	3.79 ± 0.26	0.17	3.77 ± 0.25	3.80 ± 0.31	0.62
K value (D)	44.04 ± 1.19	43.85 ± 1.44	0.49	43.87 ± 1.30	44.02 ± 1.24	0.69	43.95 ± 1.24	44.25 ± 1.26	0.34
Central foveal thickness (µm)	213.41 ± 21.08	210.38 ± 19.70	0.51	213.64 ± 24.32	212.64 ± 20.45	0.87	212.56 ± 21.28	213.79 ± 18.06	0.81
Choroidal thickness (µm)	183.35 ± 62.13	219.69 ± 66.82	0.01	123.79 ± 56.06	200.13 ± 60.52	< 0.01*	180.51 ± 61.52	248.16 ± 50.48	< 0.01*

* Indicate statistically significant (*P* < 0.05)

ACD, anterior chamber depth; C/D ratios, Cup/ disc ratios; K value, cornea curvature

**Table 4:** Mean values of parameters in eyes with and without peripheral retinal changes

Parameters	Eyes without peripheral changes(*n* = 55)	Eyes with peripheral changes(*n* = 65)	*P value*	
Gender (Female: Male)	29/26	34/31	0.96	
Age (Year)	10.71 ± 2.79	12.08 ± 3.08	0.01*	
BMI, Body mass index	17.51 ± 3.31	17.79 ± 2.95	0.62	
Spherical equivalent(D)	-8.13 ± 2.19	-9.26 ± 3.25	0.03*	
BCVA (LogMAR)	0.18 ± 0.29	0.22 ± 0.33	0.41	
IOP (mmHg)	17.23 ± 2.71	17.54 ± 2.74	0.6	
Family history	15	9	0.11	
Axial length (mm)	26.39 ± 1.20	26.92 ± 1.51	0.04*	
C/D ratios	0.30 ± 0.06	0.32 ± 0.10	0.2	
ACD	3.79 ± 0.27	3.76 ± 0.25	0.54	
K value (D)	44.17 ± 1.29	43.85 ± 1.19	0.16	
Central foveal thickness (µm)	216.54 ± 22.73	209.57 ± 18.69	0.07	
Choroidal thickness (µm)	204.69 ± 69.42	179.77 ± 59.09	0.03*	

95% CI = 95% confidence interval, OR = odds ratio

* Indicate statistically significant (*P* < 0.05)

ACD, Anterior chamber depth; C/D ratios, Cup/ disc ratios; K value, cornea curvature

### Presence of peripheral retinal changes

The findings showed that 65 eyes (54.17%) had one or more peripheral retinal changes, including retinal holes, lattice degeneration, white without pressure, snail-track degeneration, frost degeneration, retinal thinning, and peripheral pigmentary degeneration. The mean ± SD spherical equivalent refractive errors for eyes with and without peripheral retinal changes were − 9.26 ± 3.25 and − 8.13 ± 2.19 D, respectively. A Student t-test analysis showed that eyes with higher degrees of high myopia, longer axial lengths, thinner choroids, and older ages than eyes without peripheral changes. (Table 5). A binary logistic regression analysis revealed that, after controlling for axial length, both age and choroidal thickness were significantly related to the presence of peripheral retinal changes (OR = 1.194, 95% CI: 1.045–1.363, *p* = 0.009; OR = 0.993, 95% CI: 0.987–0.999, *p* = 0.022, respectively; Table 4).

**Table 5:** Logistic regression analysis to explore the factors associated with retinal changes

Parameters	B	SE	Wald	*P*	OR	95% CI of OR
Optic nerve crescent						
CT, µm	-0.01	0.004	7.105	0.008	0.99	0.983–0.997
Posterior scleral staphyloma						
CT, µm	-0.027	0.007	13.757	< 0.001	0.974	0.960–0.987
Tessellate fundus						
CT, µm	-0.018	0.005	14.756	< 0.001	0.983	0.974–0.991
Peripheral retinal changes						
age, years	0.177	0.068	6.835	0.009	1.194	1.045–1.363
CT, µm	-0.007	0.003	5.218	0.022	0.993	0.987–0.999

CT, choroidal thickness

## Discussion

More than half of the asymptomatic individuals in this study had one or two retinal changes. White without pressure and frost degeneration were the most common peripheral retinal changes, while retinal holes and lattice degeneration were important risk factors for retinal detachment in 1.67% and 15% of eyes, respectively. Tessellated fundus, optic nerve crescents, and posterior staphyloma were the most commonly found posterior retinal changes.

Tessellated fundus is considered the first stage of pathological myopia. Some tessellated fundus can progress to diffuse atrophy and macular atrophy, causing severe, irreversible visual impairment, while others remain stable at the first stage for a long time [12, 13]. Chen et al. [14] performed a meta-analysis to assess the clinical features of tessellated fundus and its relationship with myopia; the findings showed that the prevalence of tessellated fundus varies from 43 to 94.35% and that its severity is significantly associated with older ages, the male sex, long axial lengths, and thin choroids, suggesting a potential progressive pattern. Our results are consistent with these findings. Besides these characteristics and associated factors, the different locations of tessellated fundus and its relationship with myopic maculopathy have been investigated in several studies [15–17], leading to a more nuanced understanding of this unique fundus feature. However, further research is required to explore the clinical implications and underlying mechanisms. For instance, deep learning algorithms may be used for the automatic detection and categorization of clinically significant retinal lesions [18].

Optic nerve crescents are relatively stable fundus changes that do not lead to severe lesions for an extended period. We found a higher prevalence of optic nerve crescents in our study than in previous studies. Cheng et al. [6] reported that 52.5% of 12–18-year-old ethnic Chinese adolescents’ eyes had optic nerve crescents, with myopia ranging from − 6 to -17.13 D. In that study, optic nerve crescents were associated with a family history of myopia and long axial lengths. Bansal [4] reported that optic nerve crescents were present in 38.8% of eyes in children less than 10 years of age, with myopia ranging from − 6 to -25 D. In our study, eyes with optic nerve crescents had a higher degree of myopia and thinner choroids than eyes without optic nerve crescents.

In this study, we evaluated the association between posterior staphyloma and spherical equivalent refractive error, axial length, and choroidal thickness. Eyes with posterior staphyloma had significantly higher magnitudes of myopia, longer axial lengths, and thinner choroids than those without posterior staphyloma. Similar findings have been reported in previous studies. Lai et al. [7] found that 11% of their subjects’ eyes had posterior pole lesions and long axial lengths, and a high degree of myopia was associated with the presence of posterior pole lesions. Around 25% of the subjects in the Blue Mountain Eye Study were found to have posterior retinal changes; these subjects were aged 49 years or older, whereas we included subjects aged 7–16 years.

Staphyloma is a hallmark of pathologic myopia and is associated with myopic macular retinoschisis and choroidal neovascularization [3, 19, 20]. In this study, we found markedly thinned choroids. Similar findings have been reported in previous studies [21, 22]. It has been hypothesized that choroidal thinning leads to posterior scleral thinning because the inner scleral is nourished by the choroid and, thus, attenuation of the choroidal vessels may cause scleral thinning. However, this hypothesis has not yet been proven.

We also found strong associations between peripheral retinal changes and spherical equivalent refractive error, axial length, choroidal thickness, and age of the subjects. These findings are consistent with those of Chen et al.’s study [23], in which an increase in axial length was associated with lattice degeneration and retinal holes. The prevalence of lattice degeneration in our study was 14.88%; this is in agreement with the results of a Chinese study conducted in Hong Kong [7] but slightly higher than or comparable to the reported values for other ethnicities [2, 24]. Furthermore, after adjusting for axial length, we found that age and choroidal thickness were independent factors for the presence of peripheral retinal changes. High myopia-related lattice degeneration with thin choroids was critical risk factor for retinal detachment in our study. Close follow-ups of these patients might be warranted as they age.

This study has several limitations. First, the subjects were recruited from a single clinic, which may have led to selection bias, even though we selected consecutive cases. Second, we excluded patients with a history of retinal detachment, which may have led to an underestimation of the prevalence of retinal changes. Third, the sample size was relatively small, and causality could not be confirmed due to the cross-sectional study design. The strength of our study was that we comprehensively evaluated how ocular and general parameters are associated with retinal changes in 7–16-year-olds, which have not been characterized in previous studies.

In conclusion, several peripheral retinal and posterior pole changes were found in the children and adolescents included in our study. An increased risk of retinal changes was associated with high degrees of myopia, long axial lengths, thin choroids, and older ages among 7–16-year-old individuals. Therefore, for this age group of children and adolescents with high myopia, thorough fundus examinations should be performed at regular intervals.

## Data Availability

The data supporting the results reported in this article are not publicly available but can be accessed by communicating with the corresponding author.
